# Exploring the Effects of Working for Endowments on Behaviour in Standard Economic Games

**DOI:** 10.1371/journal.pone.0027623

**Published:** 2011-11-16

**Authors:** Freya Harrison, Claire El Mouden

**Affiliations:** 1 Department of Zoology, University of Oxford, Oxford, United Kingdom; 2 Nuffield College, University of Oxford, Oxford, United Kingdom; Hungarian Academy of Sciences, Hungary

## Abstract

In recent years, significant advances have been made in understanding the adaptive (ultimate) and mechanistic (proximate) explanations for the evolution and maintenance of cooperation. Studies of cooperative behaviour in humans invariably use economic games. These games have provided important insights into the mechanisms that maintain economic and social cooperation in our species. However, they usually rely on the division of monetary tokens which are given to participants by the investigator. The extent to which behaviour in such games may reflect behaviour in the real world of biological markets – where money must be earned and behavioural strategies incur real costs and benefits – is unclear. To provide new data on the potential scale of this problem, we investigated whether people behaved differently in two standard economic games (public goods game and dictator game) when they had to earn their monetary endowments through the completion of dull or physically demanding tasks, as compared with simply being given the endowment. The requirement for endowments to be ‘earned’ through labour did not affect behaviour in the dictator game. However, the requirement to complete a dull task reduced cooperation in the public goods game among the subset of participants who were not familiar with game theory. There has been some effort to test whether the conclusions drawn from standard, token-based cooperation games adequately reflect cooperative behaviour ‘in the wild.’ However, given the almost total reliance on such games to study cooperation, more exploration of this issue would be welcome. Our data are not unduly worrying, but they do suggest that further exploration is needed if we are to make general inferences about human behaviour from the results of structured economic games.

## Introduction

Cooperation – where one individual's actions increase the fitness of another – can be favoured by natural selection for two reasons. First, cooperation can increase the actor's reproductive success (i.e. cooperation confers direct fitness benefits); second, the actor can direct cooperation at individuals who also carry the cooperative gene (i.e. cooperation confers indirect fitness benefits) [1], [2]. Evolutionary explanations for cooperation, and the effects of environment and population structure on selection for cooperation, are well understood (reviewed by [1], [3]–[5]). Our own species also possesses a variety of behavioural adaptations that promote apparently selfless behaviour. These ‘proximate’ explanations (*sensu* [6], [7]; see also [8]) of sociality include a tendency toward direct (e.g. [9]) and indirect [10], [11] reciprocity and the punishment of defectors (e.g. [12], [13]). The neurological basis for cooperation has also received attention (e.g. [14]–[18]). This work helps to explain why, when people play anonymous one-shot economic games, they cooperate more than would be expected if they were purely self-interested [19]–[21].

The most common way to investigate our propensity to cooperate, reciprocate or punish is to use economic games ([8], [9], [20]–[23] and discussed by [24]). In these experiments, participants are given monetary tokens to invest in public goods or to divide with a partner in ultimatum, trust or dictator games. These tokens are later converted into real earnings. Such economic games provide important insights into the roles of reciprocity [9], [10], reputation [10], [25], punishment [12], [13], between-group competition [26], [27], negotiation [28] and fairness norms [29] in maintaining cooperation. Experiments have also demonstrated individual [18], [30], cultural [31]–[33] and sex-based [34] differences in cooperative strategies.

However, the extent to which these token-based games reflect behaviour outside the laboratory is not clear (for discussion of this issue, see [24], [35]–[38]). On a simple level, Benz & Meier [39] report that individuals make similar decisions about charitable giving both in the lab and in a natural setting. A larger study of Ethiopian forest user groups by Rustagi et al. [40] reports a correlation between the number of conditional cooperators as identified in a lab game and the success of that group in managing the real-world public good. Consistent with this, Fehr & Liebbrandt's study of Brazilian fishermen [41] showed that individuals who contribute more to the public good in a lab game use nets with larger holes, which presumably allow more immature fish to escape and breed, maintaining the real-world public good on which the participants' livelihoods depend (see also [42]). In contrast, Lamba's [43] study of Indian villagers found that an individual's behaviour in a public goods game did not predict whether or not they would exploit a real, valuable public good. In this study, play in a standard, structured economic game did not predict how selfishly people behaved in the ‘real world’.

In the real world of economic and biological markets, behavioural strategies incur real costs and benefits, in terms of effort, money, status, time, personal risk and, ultimately, Darwinian fitness. Clearly one cannot ask experimental participants to take personal risks or to incur fitness losses, but it is possible to ask participants to use their own money in games or to make participants ‘work’ for their endowment. A recent experiment [44] concluded that mean contributions to a public goods game were not affected when participants had to provide their own endowments; however, a re-analysis [45] disputed this conclusion and revealed that the proportion of ‘free riders’ – participants who contributed nothing to the public good – was actually higher among participants who used their own money to play the game. Various authors have studied how behaviour in standard games changes when the endowment level is dependent upon a person's performance in a task (usually a quiz [46]) compared with when endowments are randomly allocated. The results are varied. A recent meta-analysis concluded that, on average, participants in dictator games gave less when they had earned their endowment [21]; however, Vilares et al. [47] showed there was no difference in behaviour in trust games when endowments were either supplied gratis or earned via a physically-demanding task. Aside from quizzes, we are aware of only three experiments that imposed tangible costs whilst specifically looking at variables that are predicted to affect a person's willingness to invest effort to benefit others. Heyman and Ariely [48] used effort in a computer-based task to study the effect of reward magnitude on investment and Madsen *et al.* [49] and Harrison *et al.* [50] used a physically-demanding exercise to investigate the effect of genealogical relatedness and social proximity, respectively.

Given that these results are so varied, more work to explicitly test how earning endowments affects behaviour in standard economic games is needed. Here, we test how working to earn endowments affects behaviour in a highly structured economic game (the public goods game, PGG) and in a much simpler measure of pro-social tendency (donation to a charity in a dictator game, DG). We compared two qualitatively different tasks in our ‘earning’ condition: a time-consuming task, and a physically-demanding task. In the DG we also allocated heterogeneous endowments according to success in the task to test for any effect of endowment size on the amount donated: not only do people earn their resources in real life, but their earnings depend on their success and/or how hard they work [46]. In addition, we examined whether prior knowledge of game theory might moderate the effect of earning endowments: other authors (e.g. [51]) have shown that knowledge affects behaviour in standard games.

Participants in our experiment played either a DG or a PGG. In both cases, they were assigned to one of three treatments: monetary endowments were either given to the participant (M condition) or had to be earned via completion of a dull (T1) or physically demanding (T2) task. The dull task required participants to put pipette tips into boxes and the physically demanding task required them to squat in an isometric ski training exercise (after [49] and [50]). The DG was a one-shot game in which participants were randomly assigned to the M1, T1 and T2 conditions and told they would be given or made to earn their endowment accordingly and then have the option of donating as much or as little of the endowment as they wanted to a well-known UK charity. In the PGG, participants played five rounds of the game anonymously in groups of three. Groups were randomly assigned to the M, T1 or T2 conditions. In rounds 1, 2, 4 and 5 participants in all conditions were simply given the endowment. The nature of round 3 differed between experimental conditions: in the M condition the endowment was provided gratis as in the other rounds, but in the T1 and T2 conditions endowments in round 3 had to be earned by completing the tasks described above. Embedding the T1 and T2 conditions in a sequence of M rounds allowed us to a) test for any pre-treatment differences between participants in the three conditions and correct for them if necessary; b) test for between-subjects differences in behaviour across the three conditions; and c) test for any ‘carry-over’ effects of working for endowments when participants returned to being provided with endowments in rounds 4 and 5. Because we wanted to compare levels of cooperation between our treatment groups, we imposed a game structure that, on the whole, favours cooperation. Otherwise, differences between conditions may be obscured by a general decay of cooperation towards a selfish optimum. We achieved this by introducing an element of between-group competition, a structure known to favour cooperation [1], [26], [27], [52] – participants were told that if they were in one of the three groups that earned the highest total amount of money, they would each be awarded a voucher for a major online retailer.

## Results

### 1. Task validation and dictator game

90 participants took part in the DG, 30 being assigned to each of the three treatments. We used questionnaires during the DG to ascertain that the tasks we used were perceived differently from one another, and from the M condition. Approximately one-third of participants felt that they owned the money they had been given, and this did not vary across treatments (10/30 for M, 12/30 for T1 and T2: χ^2^_2_ = 0.378, *p* = 0.828). None of the participants in the M condition felt that they had earned their endowment, while 19/30 and 17/30 participants in the T1 and T2 conditions respectively felt it had been earned. The proportion of participants who felt they had earned their money was not significantly different between the two task treatments (χ^2^_1_ = 0.28, *p* = 0.598). Participants were more likely to consider the pipette task dull, as compared with the squatting task (20/30 vs. 10/30 participants in the two conditions said they found the task dull: χ^2^_1_ = 6.67, *p* = 0.010). The squatting task was more likely than the pipette tip task to be considered difficult (26/30 vs. 8/30 participants: χ^2^_1_ =  21.99, *p*<0.001).

**Figure 1:**
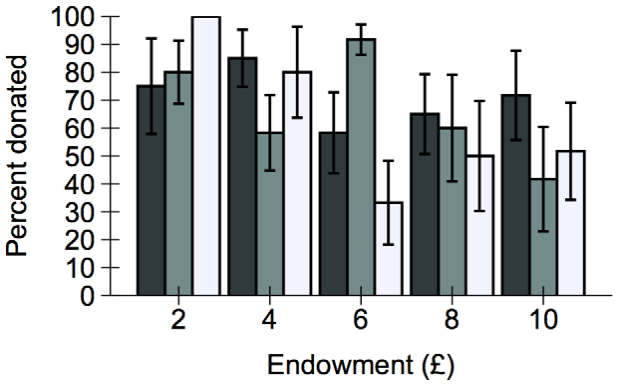
Proportion of endowment donated to Oxfam in the dictator game. Black bars show participants who were given money (M), grey bars participants who performed the pipette tip task (T1) and white bars participants who performed the squatting task (T2). Bars show mean percentage donation ± one standard error. The proportion of endowment donated did not differ between treatments (F_2,15_ = 0.02, *p* = 0.977), however people receiving larger endowments donated a smaller proportion to charity (F_1,58_ = 6.66; *p* = 0.012).

We tested whether the proportion of endowment donated by each participant was affected by sex, age, knowledge of game theory, treatment and endowment size using a logit transformed GLM. The best model, as defined using Akaike's Information Criterion (AICc: [53]) included only the main effects of these terms. The proportion of endowment donated did not differ between treatments (F_2,15_ = 0.02, *p* = 0.977, Figure 1), but there was a slight negative correlation between endowment size and proportion donated (F_1,58_ = 6.66; *p* = 0.012, partial η^2^ = 0.103). This is likely due to the very small and potentially ‘throwaway’ amounts of money used at the lower end of our scale. There was no effect of sex (F_1,58_ = 0.48; *p* = 0.490), age (F_1,58_ = 0.38; *p* = 0.542) or knowledge of game theory (F_1,58_ = 0.20; *p* = 0.660).

### 2. Public goods game

**Figure 2:**
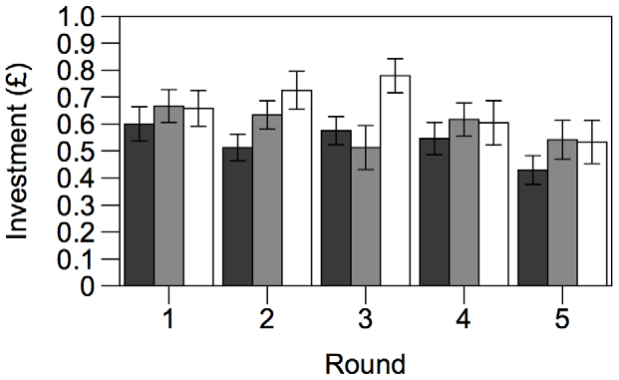
Investment in the public goods game. Raw data (means ± standard error) of investment by participants in the three treatment conditions in rounds 1-5 of the game.

72 participants took part in the PGG, 24 being assigned to each of the three conditions. The raw data are plotted in Figure 2. We first verified that our M condition produced results comparable with the existing literature on PGGs. In round 1, participants invested an average of 62.5±2.24% of their endowment. GLMM showed that the trend in investment over the five rounds was best described by a linear function, with investment showing a shallow decline over time (F_1,72_ = 5.82, *p* = 0.018; slope  =  -3.1±1.28; partial η^2^ = 0.075). This is broadly consistent with results reported by other authors [20], [54]–[57]. The slope differed significantly between individual participants (F_16,72_ = 3.05, *p* = 0.001). We calculated the fitted slopes for each individual participant from this model and used GLMM to test for significant effects of sex, age and knowledge of game theory on the slope. No such effects were found (*p* ≥ 0.105).

We then verified that there was no effect of condition on investment in rounds 1 and 2, when participants in all three conditions played the same game. Neither condition nor round were significant as main effects (F_2,21_ = 2.96, *p* = 0.245 and F_1,69_ = 0.27, *p* = 0.602, respectively) and neither was their interaction (F_2,69_ = 1.71, *p* = 0.188).

**Figure 3:**
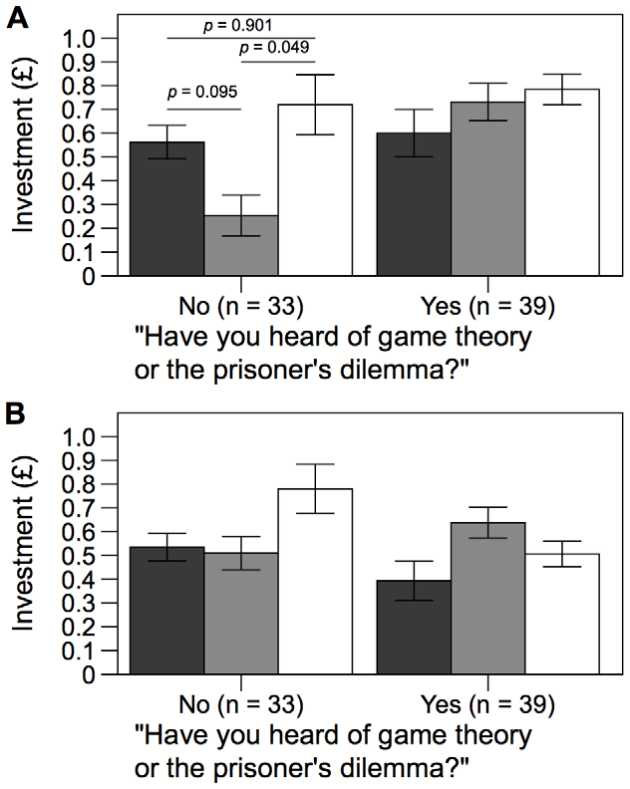
Investment in the public goods game. a) In round 3, investment levels were constant across the three conditions among participants who had heard of game theory, but varied across conditions among participants who had not heard of game theory (F_1,65_ = 3.83, *p* = 0.027). All pairwise comparisons of conditions among participants who had heard of game theory were non-significant (*p*>0.6). b) Average investment in round 4 and 5. Investment did not differ between M, T1 and T2 conditions among participants who were unfamilar with game theory, nor between participants who were familiar with game theory (*p*>0.3). Bars show mean ± standard error; black bars show M condition, grey bars T1 and white bars T2. *p*-values for pairwise comparisons are from Tukey post-hoc tests.

Having thus satisfied ourselves that there were no pre-treatment differences between our experimental groups, we analysed investment behaviour in round 3, when conditions actually varied. We were interested in potential effects of condition, sex, age, knowledge of game theory and interactions between these variables. The best model, as defined using Akaike's Information Criterion (AICc: [53]), is shown in Table 1. There was no main effect of condition (F_2,65_ = 2.82, *p* = 0.067), which is not consistent with our main hypothesis that earning investments alters behaviour. However, there was a significant condition x game theory interaction (F_2,65_ = 3.83, *p* = 0.007). As illustrated in Figure 3a, this was driven by a difference between the two task conditions among participants who were not familiar with game theory: in this subset of participants, investment following the dull, pipette tip task (T1) was lower than investment following the physical task (T2; Tukey post-hoc comparison, *p* = 0.049). Further, investment following T1 was lower among participants who were unfamiliar with game theory as compared with those who were familiar with game theory (Tukey post-hoc comparison, *p* = 0.003). There was also a significant effect of sex, such that women gave slightly more to the public good than men (F_2,65_ = 8.44, *p* = 0.005).

**Table 1:** Analysis of variance for individual investment in a) round 3 and b) rounds 4 and 5 of the public goods game.

a)			
**Source**	**DF**	**F**	***p***
condition	2	2.81	0.067
sex	1	8.44	0.005
game theory	1	8.00	0.006
condition x game theory	2	3.83	0.027

b)			
**Source**	**DF**	**F**	***p***
condition	2	1.58	0.211
sex	1	5.45	0.021
game theory	1	1.47	0.228
condition x game theory	2	3.93	0.022

We then sought to determine whether condition influenced behaviour in the two rounds after the treatment round, where participants in all conditions once again played the M game. Again, there was no main effect of condition (F_2,136_ = 1.58, *p* = 0.211), knowledge of game theory (F_1,136_ = 1.47, *p* = 0.228) or round (F_1,136_ = 2.59, *p* = 0.110) but there was a significant condition x game theory interaction (F_2,136_ = 3.93, *p* = 0.022). As illustrated in Figure 3b this interaction was due to a difference between investment following T2 when comparing participants who were familiar or unfamiliar with game theory (Tukey post-hoc comparison, *p* = 0.046); post-hoc comparisons between conditions within each subset of participants were not significant (*p*>0.3). Therefore the divergent reaction to T1 and T2 among participants unfamiliar with game theory disappeared in rounds 4 and 5.

## Discussion

The tasks we used appear well suited for experimental use: they were easy to implement and were viewed differently by participants. We found no effect of performing either task to earn endowments on behaviour in the DG. However, we found that people who completed the dull task and were unfamiliar with game theory cooperated less than the control and difficult task groups in the public goods game. Our results do not undermine the standard uses of cooperation games. We did not find any strong evidence against such games having the ability to explain human behaviour more generally. However, our results do suggest that we should be aware that there are likely to be behavioural differences between participant pools drawn from people cognisant of game theory and people unaware of this field (see [58]).

Participants who had heard of game theory behaved differently in the PGG from those who had not: this is not entirely surprising [51], but its interaction with performance of a dull task is interesting. Further experimental work could usefully test the robustness of this effect and, if it stands up to more explicit scrutiny, address it in more detail. We hypothesise that, among participants who are familiar with game theory, the framing effects of presenting them with “an economic game” induced strategic behaviour. In our design, inter-group competition for an additional payoff (shopping vouchers) went some way to aligning individual and group interests and it is possible that people who are familiar with game theory were more likely to focus on this potential gain and play strategically, whereas people who were not behaved more “emotionally” after performing the dull task. It is interesting that the physical task did not have this effect and it is possible that this instead was interpreted as a “team-building” exercise. We noticed that participants appeared to be focussed on their own pipette tip boxes and not engaging with one another, whereas they seemed much more communicative in the physical task. In future it would be useful to ask participants explicitly about their feelings towards the tasks and towards their team-mates while performing tasks [52], [59]. Further, we did not test the extent of our participants' knowledge of game theory. In any future work, a more thorough assessment of how deeply participants understand game theory would facilitate a better exploration of how knowledge of game theory influences behaviour.

Other authors have reported [34] that different game structures can lead to different levels of cooperation and earning seems to have different effects in dictator and trust games [21], [47]. It is unclear why a) our two different tasks had divergent effects on behaviour in our PGG and b) a comparable effect was not present in the DG. While the framing [57] of actions in games has received a lot of general attention (e.g. [60]–[65]), the differences in framing and interpretation of different standard games would benefit from empirical research (e.g. [66]). In our case, the explanation may simply be that the PGG by nature is more complex than the DG, requiring more organisation and a more structured environment for participants. A separate but related issue is the extent to which a participant's ‘cultural baggage’ may affect their interpretation of games and their behaviour in the lab [58], [67]. In general, it is essential to understand what people are thinking, what features of the experimental environment they find salient and how they perceive different games.

We have presented initial findings that behaviour in a PGG can be affected by requiring participants to earn their endowment by means of a dull task, provided that those participants are unfamiliar with game theory. Further exploration of how the model tasks we employed affect economic/cooperative behaviour could prove very useful for future experimental work. For example, it would be interesting to test how people perceive co-players in games with and without tasks (see [52]). More importantly, we recommend a more thorough exploration of the effects of these tasks on behaviour in a larger study population – perhaps a population that is expected to be naïve with regard to game theory – and with different game structures.

## Methods

### 1. Ethics statement

This study was designed in accordance with the ethical guidelines provided by the University of Oxford and the British Psychological Society and received ethical approval from the University of Oxford's Social Sciences and Humanities Interdepartmental Research Ethics Committee (reference: SSD/CUREC1/10-284). Participants were recruited from students and staff at the University of Oxford – mainly those working or attending lectures in the Department of Zoology, though a minority of participants were recruited by snowball sampling. All participants provided written informed consent. Participants were advised that part of the experiment (T2 condition, see below) was not suitable for people with back or knee problems; any participants who said this would not be suitable for them were assigned to another condition. Information supplied to participants and questionnaires used are provided as Supporting Information S1.

### 2. Task validation and dictator games

90 participants (44 female) took part in this experiment, which was framed as “a study of people's feelings about money and giving to charity.” Participant age ranged from 18 to 45 years (mean 21.9±0.54 years). Participants were randomly assigned into groups of 5 and each group randomly assigned to one of three treatment conditions: Money (M); Task 1 (T1); or Task 2 (T2). 30 participants took part in each condition. In the M condition, each group member was randomly assigned £2, £4, £6, £8 or £10. In the T1 condition, each group member was given four empty pipette tip boxes (each designed to hold 96 200µl pipette tips) and asked to fill them with tips as fast as possible; the fastest person was assigned £10, the next £8, and so on down to the slowest person who received £2. In the T2 condition, participants were asked to squat in an isometric ski training position for as long as they could. The group member who squatted the longest received £10, the second longest £8 and so on. Each group of five performed their task simultaneously, i.e. there was no privacy within groups. Groups were, however, separated from one another; groups tested at the same time were separated by screens. Participants received their endowment in a numbered money bag. The endowment was supplied divided into tenths (achieved by using appropriate denominations and/or taping coins together).

Median age did not vary across treatments (Kruskal Wallis Test: H = 4.27, D.F.  = 2, *p* = 0.118, n = 89 as one participant did not give their age). The proportion of females did not vary across treatments (χ^2^_2_ = 3.29, *p* = 0.193, n = 88). 64 participants said that they had previously heard of game theory: males were more likely to have heard of game theory than females (χ^2^_1_ = 6.07, *p* = 0.014, *n* = 81), but overall the proportion of participants who had heard of game theory did not vary across treatments (χ^2^_2_ = 77, *p* = 0.682, *n* = 83).

After receiving their endowment, participants answered a short questionnaire asking about demographic information and their feelings about the endowment. Specifically, they were asked whether they agreed or disagreed with the following statements: “I earned the money I was given”; “the money I was given belonged to me”; “the task I did was dull” and “the task I did was difficult”. Participants were instructed to go behind a screen, remove as much of the endowment (in tenths) as they wanted to keep, re-seal the bag and place it in a donation box. Amounts donated were linked to unique participant numbers, maintaining anonymity. Participants were informed that all donations went to Oxfam (http://www.oxfam.org.uk; this charity has long been a household name in the United Kingdom and has no political or religious affiliation). The charity's logo was used on posters in the experimental area to reassure participants that money would really be donated. A total of £337.80 was donated to Oxfam by participants in this experiment.

We used general linear mixed models (GLMM) to determine whether the proportion of the endowment donated was affected by treatment, sex, age, knowledge of game theory or endowment size, including group as a random factor. The results given are for analyses that exclude nine participants who did not state their sex and/or knowledge of game theory; including these participants did not change the results. Proportion of endowment donated was logit transformed to meet the assumptions of homoscedasticity and normality of error.

### 3. Public goods games

72 participants (37 female) took part in this experiment. Participant age ranged from 18 to 40 (mean 22.2±0.44 years). 39 participants said that they had previously heard of game theory and the frequency of participants who had heard of game theory was not different for females and males (χ^2^_1_ = 0.93, *p* = 0.33). Because significant written information was provided on game structure and payoffs, we verified that all participants spoke fluent English. Before commencing the experiment, participants were given a table showing examples of possible team investment patterns and payoffs and asked to verify that they understood the game; participants were given the opportunity to ask questions before the game began to ensure their understanding.

Participants were randomly assigned into 24 teams of three people and each team assigned to either the money (M) or task (T1, T2) conditions. 2-3 teams played the game in each experimental session and individuals were randomly assigned to teams, i.e. they did not know which of the other people in the room were in their team. Teams remained fixed over the course of the game. In the M condition, participants played five rounds of a simple public goods game: in each round, each participant received an endowment of £1, a fraction of which (0, 0.2, 0.4, 0.6, 0.8 or 1) they could contribute to a team investment. Participants kept any money they did not invest. In the T1 condition, rounds 1, 2, 4 and 5 of the game were identical to the M game, but in round 3, participants were given four empty pipette tip boxes and told that they would earn that round's endowment in return for filling the boxes with tips. The T2 condition was identical to the T1 condition except that participants earned their endowment in round 3 by squatting in an isometric ski training exercise for 45 seconds. In T1 and T2, all participants completed the task. In each round, the total invested by a team was multiplied by 1.5 and divided equally between the team members. Participants were told that the number of rounds in the game, and the instance of the labour round, would be determined randomly. This was to remove the risk of a “last round” effect and/or of participants using backward induction to determine the best investment strategy [54]. Participants were told their individual payoff after each round and their total payoff at the end of the game.

Because we wanted to compare levels of cooperation between our treatment groups, we chose to impose a game structure that, on the whole, favoured cooperation. Otherwise, differences between conditions may have been obscured by a general decay of cooperation towards a selfish optimum regardless of condition. Inter-group competition goes some way to aligning individuals' selfish interests with those of the group, favouring cooperation on both evolutionary [1] and behavioural [26], [27], [52] timescales. We therefore imposed inter-group competition by awarding vouchers for a major online retailer for the three groups which gained the highest total earnings across all five rounds of the game and across all conditions.

Data on per-round individual and team investment were analysed using GLMM. Participant was declared as a random factor nested in team, which was itself a random factor nested in condition. Round was coded as categorical variable to allow for post-hoc pairwise comparisons between rounds and/or groups. When all rounds in the M condition were analysed together, individual investment was square root transformed to meet model assumptions.

## Supporting Information

Supporting Information S1 Information and instructions provided to participants in the DG and PGG.
(DOC)

## References

[1] Frank SA (1998). Foundations of Social Evolution..

[2] Hamilton WD (1964). The genetical evolution of social behaviour I & II.. J Theor Biol.

[3] Gardner A, Foster KR, Korb J, Heinze J (2008). The evolution and ecology of cooperation - history and concepts.. Ecology of Social Evolution: Springer.

[4] Sachs JL, Mueller UG, Wilcox TP, Bull JJ (2004). The evolution of cooperation.. Quarterly Review of Biology.

[5] West SA, Griffin AS, Gardner A (2007). Evolutionary explanations for cooperation.. Current Biology.

[6] Mayr E (1961). Cause and effect in biology.. Science.

[7] Tinbergen N (1963). On aims and methods of ethology.. Zeitschrift fur Tierpsychologie.

[8] West SA, El Mouden C, Gardner A (2011). Sixteen common misconceptions about the evolution of cooperation in humans.. Evolution and Human Behavior.

[9] Gachter S, Herrmann B (2009). Reciprocity, culture and human cooperation: previous insights and a new cross-cultural experiment.. Philosophical Transactions of the Royal Society B-Biological Sciences.

[10] Wedekind C, Milinski M (2000). Cooperation through image scoring in humans.. Science.

[11] Milinski M, Semmann D, Krambeck H-J (2002). Reputation helps solve the ‘tragedy of the commons’.. Nature.

[12] Fehr E, Fischbacher U (2004). Third-party punishment and social norms.. Evolution and Human Behavior.

[13] Fehr E, Gachter S (2002). Altruistic punishment in humans.. Nature.

[14] Behrens TE, Hunt LT, Rushworth MF (2009). The computation of social behavior.. Science.

[15] Stevens JR, Cushman FA, Hauser MD (2005). Evolving the psychological mechanisms for cooperation.. Annual Review of Ecology, Evolution, and Systematics.

[16] Suzuki S, Niki K, Fujisaki S, Akiyama E (2011). Neural basis of conditional cooperation.. Social Cognitive and Affective Neuroscience.

[17] Tankersley D, Stowe CJ, Huettel SA (2007). Altruism is associated with an increased neural response to agency.. Nat Neurosci.

[18] Wischniewski J, Windmann S, Juckel G, Brüne M (2009). Rules of social exchange: game theory, individual differences and psychopathology.. Neurscience and Behavioral Reviews.

[19] Fehr E, Fischbacher U (2003). The nature of human altruism.. Nature.

[20] Ledyard JO, Kagel JH, Roth AE (1995). Public Goods: A Survey of Experimental Research.. The Handbook of Experimental Economics.

[21] Engel C (2010). Dictator games: a meta study.. Experimental Economics.

[22] Fehr E, Rockenbach B (2004). Human altruism: economic, neural, and evolutionary perspectives.. Current Opinion in Neurobiology.

[23] Hammerstein P, Hagen EH (2005). The second wave of evolutionary economics in biology.. Trends in Ecology & Evolution.

[24] Falk A, Heckman JJ (2009). Lab experiments are a major source of knowledge in the social sciences.. Science.

[25] Sommerfeld RD, Krambeck H-J, Semmann D, Milinski M (2008). Gossip as an alternative for direct observation in games of indirect reciprocity.. PNAS.

[26] Puurtinen M, Mappes T (2009). Between-group competition and human cooperation.. Proceedings of the Royal Society B: Biological Sciences.

[27] West SA, Gardner A, Shuker DM, Reynolds T, Burton-Chellow M (2006). Cooperation and the scale of competition in humans.. Current Biology.

[28] Cardenas JC, Stranlund J, Willis C (2000). local environmental control and institutional crowding-out.. World Development.

[29] Mellers BA, Haselhuhn MP, Tetlock PE, Silva JC, Isen AM (2010). Predicting behavior in economic games by looking through the eyes of the players.. Journal of Experimental Psychology: General.

[30] Kurzban R, Houser D (2001). Individual differences in cooperation in a circular public goods game.. European Journal of Personality.

[31] Gächter S, Herrmann B, Thöni C (2010). Culture and cooperation.. Philosophical Transactions of the Royal Society B: Biological Sciences.

[32] Henrich J, Boyd R, Bowles S, Camerer C, Fehr E (2005). ‘Economic man’ in cross-cultural perspective: behavioural experiments in 15 small-scale societies.. Behavioural and Brain Sciences.

[33] Tracer DP (2003). Selfishness and fairness in economic and evolutionary perspective: an experimental economic study in Papua New Guinea.. Current Anthropology.

[34] Kümmerli R, Colliard C, Fiechter N, Petitpierre B, Russier F (2007). Human cooperation in social dilemmas: comparing the Snowdrift game with the Prisoner's Dilemma.. Procedings of the Royal Society B: Biological Sciences.

[35] Kümmerli R, Burton-Chellow M, Ross-Gillespie A, West SA (2010). Resistance to extreme strategies, rather than prosocial preferences, can explain human cooperation in public goods games.. PNAS.

[36] Hagen EH, Hammerstein P (2006). Game theory and human evolution: A critique of some recent interpretations of experimental games.. Theoretical Population Biology.

[37] Trivers R (2006). Mutual benefits at all levels of life (review of Genetic and Cultural Evolution of Cooperation, MIT Press 2003, P Hammerstein, ed).. Science.

[38] Levitt SD, List JA (2007). What do laboratory experiments measuring social preferences reveal about the real world?. The Journal of Economic Perspectives.

[39] Benz M, Meier S (2008). Do people behave in experiments as in the field? Evidence from donations.. Experimental Economics.

[40] Rustagi D, Engel S, Kosfeld M (2010). Conditional cooperation and costly monitoring explain success in forest commons management.. Science.

[41] Fehr E, Leibbrandt A (2011). A field study on cooperativeness and impatience in the tragedy of the commons.. Journal of Public Economics.

[42] Carpenter J, Seki E (2010). Do social preferences increase productivity? Field experimental evidence from fishermen in Toyama Bay.. Economic Inquiry.

[43] Lamba S, Mace R (2011). Demography and ecology drive variation in cooperation across human populations.. PNAS.

[44] Clark J (2002). house money effects in public good experiments.. Experimental Economics.

[45] Harrison G (2007). House money effects in public good experiments: comment.. Experimental Economics.

[46] Cherry TL, Kroll S, Shogren JF (2005). The impact of endowment heterogeneity and origin on public good contributions: evidence from the lab.. Journal of Economic Behavior & Organization.

[47] Vilares I, Dam G, Kording K (2011). Trust and reciprocity: are effort and money equivalent?. PLoS ONE.

[48] Heyman J, Ariely D (2004). Effort for payment: a tale of two markets.. Psychological Science.

[49] Madsen EA, Tunney RJ, Fieldman G, Plotkin HC, Dunbar RI (2007). Kinship and altruism: a cross-cultural experimental study.. British Journal of Psychology.

[50] Harrison F, Sciberras J, James R (2011). Strength of social tie predicts cooperative investment in a human social network.. PLoS ONE.

[51] Frank RH, Gilovich T, Regan DT (1993). Does studying economics inhibit cooperation?. The Journal of Economic Perspectives.

[52] Burton-Chellew M, Ross-Gillespie A, West SA (2010). Cooperation in humans: competition between groups and proximate emotions.. Evolution and Human Behavior.

[53] Hurvich CM, Tsai C-L (1989). Regression and time series model selection in small samples.. Biometrika.

[54] Camerer CF (2003). Behavioral Game Theory..

[55] Ostrom E (2000). Collective action and the evolution of social norms.. The Journal of Economic Perspectives.

[56] Zelmer J (2003). Linear Public Goods Experiments: A Meta-Analysis.. Experimental Economics.

[57] Chaudhuri A (2011). Sustaining cooperation in laboratory public goods games: a selective survey of the literature.. Experimental Economics.

[58] Henrich J, Heine SJ, Norenzayan A (2010). Most people are not WEIRD.. Nature.

[59] Hertel G, Neuhof J, Theuer T, Kerr NL (2000). Mood effects on cooperation in small groups: Does positive mood simply lead to more cooperation?.. Cognition & Emotion.

[60] Tversky A, Kahneman D (1981). The framing of decisions and the psychology of choice.. Science.

[61] Andreoni J (1995). Warm-Glow versus cold-prickle: the effects of positive and negative framing on cooperation in experiments.. The Quarterly Journal of Economics.

[62] Cookson R (2006). Framing effects in public goods experiments.. Experimental Economics.

[63] Levin IP, Schneider SL, Gaeth GJ (1998). All frames are not created equal: a typology and critical analysis of framing effects.. Organizational Behavior and Human Decision Processes.

[64] Willinger M, Ziegelmeyer A (1999). Framing and cooperation in public good games: an experiment with an interior solution.. Economics Letters.

[65] Dufwenberg M, Gaechter S, Hennig-Schmidt H (2011). The framing of games and the psychology of play.. Games and Economic Behavior:.

[66] Larrick RP, Blount S (1997). The claiming effect: Why players are more generous in social dilemmas than in ultimatum games.. Journal of Personality and Social Psychology.

[67] Henrich J, Boyd R, Bowles S, Gintis H, Fehr E (2004). Foundations of Human Sociality: Economic Experiments and Ethnographic Evidence in Fifteen Small-Scale Societies..

