# Supplementary material for: Exploring the Effects of Working for Endowments on Behaviour in Standard Economic Games
Source: PLoS One. 2011 Nov 16;6(11):e27623. doi: 10.1371/journal.pone.0027623 (PMC3217995; doi:10.1371/journal.pone.0027623)
Supplement: Supporting Information S1 — Information and instructions provided to participants in the DG and PGG. (DOC) [file pone.0027623.s001.doc]

**Supplementary information S1**: ***Exploring the effects of working for endowments on behaviour in standard economic games***

Freya Harrison & Claire El Mouden

Department of Zoology, University of Oxford, South Parks Road, Oxford OX1 3PS

1. Information given to participants prior to participating in the dictator game

2. Instructions read to participants in the dictator game

3. Questionnaire used in the dictator game

4. Information provided to participants in the public goods game

**1. Information given to participants prior to participating in the dictator game**

**We are conducting a study of people’s feelings about money and giving to charity**

**In the study, we will give you money and ask you to complete a short questionnaire**

**You will then be able to choose whether to give some or all of your money to Oxfam**

**Some of you will be asked to perform a task in order to earn the money we will give you**

*One of these tasks involves squatting in a cross-country ski-training position as in the photo. If you cannot safely perform this task (e.g. because you have back or knee problems), let us know and we will not assign you to this task*

**Your decisions will remain completely anonymous**

**All money donated to Oxfam will be passed on to Oxfam once the study is complete**

This study is led by Dr Freya Harrison (freya.harrison@zoo.ox.ac.uk)

It has been approved by the Social Sciences & Humanities Inter-Divisional Research Ethics Committee (ref no SSD/CUREC1/10-284)

**2. Instructions read to participants in the dictator game**

M condition

We have randomly assigned one of you £10, one of you £8, one of you £6, one you £4 and one of you £2.

T1 condition

You each have four empty pipette tipboxes. We would like you to fill them with tips from the bags. In return for this task, you will earn money. The first person to fill all their boxes will earn £10, the next £8, the next £6, the next £4 and the last £2.

T2 condition

We are going to ask you to squat in this cross-country ski training position [INDICATE POSTER AND DEMONSTRATE]. In return for this task, you will earn money. The first person who squats for the longest time will earn £10, the next £8, the next £6, the next £4 and the last £2.

All conditions

The money is in a numbered bag which we will give to you now; the total amount is written on the bag for you to see. Please open your bag and look at how much you have. The money is yours to keep. We have also given you a questionnaire which asks a few demographic questions and also asks about your feelings towards this money. We would now like you to fill in the questionnaire and fold it over. When you have finished, we will invite you one person at a time to go behind this screen. Please put your folded questionnaire into the box marked ‘questionnaires.’ There is also a donation box for Oxfam. You have the option of donating the money we gave you to Oxfam by putting it in this box. You may give none, some or all of your money to Oxfam as you choose; please note we have divided the money into tenths and for some amounts this means we have taped coins together. Please do not un-tape your coins. Simple remove as much money as you wish to keep for yourself, put it in your pocket or wallet, seal the remaining money in the bag (or seal the empty bag) and post it in the box marked ‘Oxfam.’ You may then leave the study area.

**3. Questionnaire used in the dictator game**

***Participant questionnaire: please write in or circle the appropriate answers.***

**Participant Number: ____________ Sex M / F Age ____________**

**How would you describe your ethnicity? ________________________________**

**Have you ever heard of game theory or the prisoner’s dilemma? Yes / No**

***Do you agree or disagree with the following statements?***

**I earned the money I was given Agree / Disagree**

**I owned the money I was given Agree / Disagree**

**The task I did was dull Agree / Disagree**

**The task I did was difficult Agree / Disagree**

**4. Information provided to participants in the public goods game**

**General information**

You have been randomly assigned into groups of three people. The other people in your group could be sitting anywhere in the room and you will not have the opportunity to find out who is in your group.

We will play a number of rounds of an investment game. In each round, you will have the opportunity to invest in a group venture.

Each round will take the form of either a money game or a group task game. The form each round takes will be randomly determined.

In the money game you will be given an endowment of money which you may choose to invest in a group account with the chance of earning more money, or keep for yourself; in the task game you will invest time in a physical task in return for money, which you may either invest in the group or keep for yourself.

In each round of investment, the total investment made by the members of a group will be multiplied by 1.5 and divided equally between the members of that group. The number of rounds of investment will be determined randomly at the start of the game.

There are other groups of people taking part in this experiment. At the end of the experiment, the members of the group that earned the highest total payoff will receive a bonus in the form of an iTunes or Amazon voucher.

**Money Game**

You have been given £1. In each round of the game, you have the option to invest 0, 10, 20, 30, 40, 50, 60, 60, 70, 80, 90 or 100p in your group’s account. Any money that you do not invest in the group account, you may keep for yourself. By investing in the group account, you may earn more money, or lose money, depending on what the other people in your group contribute. Examples of investments and payoffs are given in the table provided. At the end of the round, you will be told your payoff and given a further endowment of £1 for the next round.

.

**Group task game**

In each round of the game, you will be asked to squat in a cross-country ski training exercise for 45 seconds. This is designed to be physically demanding, but as long as you have no back, knee or joint problems carries no significant risks. Your effort in this task earns you £1. You will then have the option of investing 0, 10, 20, 30, 40, 50, 60, 60, 70, 80, 90 or 100p of this money in your group’s account. Any money that you do not invest in the group account, you may keep for yourself. By investing in the group account, you may earn more money, or lose money, depending on what the other people in your group contribute. Examples of investments and payoffs are given in the table provided.

At the end of the game, you will be told your total earnings. This money is yours to keep: you will receive it in cash soon after the experiment and we will ask you to sign a receipt for it. When we have finished this experiment, we will contact the members of the group who earned the highest group total payoff and they will each receive an iTunes or Amazon voucher.

| **Your investment** | **Investment by other two group members** | **Total in group account for this round** | **Multiplied group total** | **Each group member receives** | **Your gain or loss** |
| --- | --- | --- | --- | --- | --- |
| 100p | 100p, 100p | £3.00 | £4.50 | £1.50 | + 50p |
| 100p | 100p, 0p | £2.00 | £3.00 | £1.00 | 0 |
| 0p | 100p, 100p | £2.00 | £3.00 | £1.00 | + £1.00 |

This project has been approved by the University of Oxford Inter-Divisional Research Ethics Committee (Social Sciences). Ref: SSD/CUREC1/10-284. Lead researcher: Dr Freya Harrison (email freya.harrison@zoo.ox.ac.uk).
